# Molecular characterization and expression analysis of the *remorin* genes in tomato (*Solanum lycopersicum* L.)

**DOI:** 10.3389/fpls.2023.1175153

**Published:** 2023-05-09

**Authors:** Hui Li, Xiao Wang, Yue Zhuo, Shuisen Chen, Jingwei Lin, Hui Ma, Ming Zhong

**Affiliations:** Key Laboratory of Agricultural Biotechnology of Liaoning Province, College of Biosciences and Biotechnology, Shenyang Agricultural University, Shenyang, China

**Keywords:** tomato, *remorin*, gene family, genome-wide analysis, expression pattern

## Abstract

Remorin (REMs) are plant-specific and plasma membrane-associated proteins that play an essential role in the growth and development of plants and adaptations to adverse environments. To our knowledge, a genome-scale investigation of the *REM* genes in tomato has never been systematically studied. In this study, a total of 17 *SlREM* genes were identified in the tomato genome using bioinformatics methods. Our results demonstrated that the 17 members of SlREM were classified into 6 groups based on phylogenetic analysis and unevenly distributed on the eight chromosomes of tomato. There were 15 *REM* homologous gene pairs between tomato and *Arabidopsis*. The *SlREM* gene structures and motif compositions were similar. Promoter sequence analysis showed that the *SlREM* gene promoters contained some tissue-specific, hormones and stress-related cis-regulatory elements. Expression analysis based on qRT-PCR (Real-time quantitative PCR) analysis showed that *SlREM* family genes were were differentially expressed in different tissues, and they responded to ABA, MeJA, SA, low-temperature, drought and NaCl treatments. These results potentially provide relevant information for further research on the biological functions of *SlREM* family genes.

## Introduction

Remorin proteins are a plant-specific and plasma membrane-associated proteins (Raffaele et al., 2013), which are found in gymnosperms, angiosperms, pteridophytes and bryophytes (Checker and Khurana, 2013). The first remorin protein with predicted structure was derived from a cDNA library of potato leaves, with predicted relative molecular weight 21.769 KD, containing 198 amino acids, rich in glutamic acid and lysine, 11 of the first 50 amino acids in the N-terminal are proline, and a coiled-coil domain in the C-terminal is predicted (Reymond et al., 1996; Pawson et al., 1995). The C-terminus of Remorin protein is a very conserved coiled-coil with amino acid residues ranging from 70 to 80 (Reymond et al., 1996), and the N-terminus amino acid residues vary greatly between species (Marín and Ott, 2012). A specific short sequence in the C-terminus of remorin protein mediates the binding to plasma membrane (PM), which is called REM-CA (Perraki et al., 2012; Raffaele et al., 2013). REM-CA is an essential region for oligomerization and is directly involved in the interactions of other proteins (Marín and Ott, 2012; Tóth et al., 2012). The amino acid sequence composition and length of the N-terminal part of Remorin protein change significantly (Bariola et al., 2004), which determines the complex function of remorin protein. The N-terminal disordered region of remorin protein in plants can be used as a signal element for protein interactions. According to the structural diversity of the N-terminal, the remorin family members have been classified into 6 groups according to their functions (Raffaele et al., 2007).

Remorins are very important for plant growth and development. The rice remorin gene *OsREM4.1*, which is specifically localized to the plasma membrane and plasmodesmata (Gui et al., 2014; Gui et al., 2016). Remorin protein encoded by GSDl is attached to PM mediated by S-acylation and overexpression of GSDl leads to increased remorin protein, carbohydrate accumulation in leaves, reduced soluble sugar content and reduced rice grain size (Gui et al., 2015). The *LONG PANICLE1*(*LP1*) gene encodes a remorin protein, which affects panicle development in rice (Liu et al., 2016). In tomato, *SlREM* positively regulates fruit ripening by affecting the biosynthesis of ethylene and lycopene (Cai et al., 2018).

Abiotic stress is a major environmental factor and quality that adversely affects plant growth and productivity. For example, plants are often exposed to various environmental abiotic stresses, including drought, soil salinity, and low temperature (Gong et al., 2020). Transcriptome and proteome data analysis showed that remorin proteins can respond to various abiotic stresses, including drought, salt stress and low-temperature conditions in *Arabidopsis* and rice (Bray, 2002; Reddy et al., 2002; Nohzadeh et al., 2007). The expression of *MiREM* gene was induced by salt and dehydration stresses in mulberry leaves. Overexpression of *MiREM* gene in *Arabidopsis* enhances drought and salt-stress tolerance (Checker and Khurana, 2013). The expression of *SiREM6* increased after high-salt, low-temperature and exogenous ABA conditions in foxtail millet (*Setaria italica*) (Li et al., 2013). Overexpression of *SiREM6* increased the tolerance of *Arabidopsis* to high salt during germination and seedling stages (Yue et al., 2014). *DaCBF7* from *Deschampsia antarctica* in transgenic rice plants upregulates remorin gene expression, thus improving cold tolerance (Byun et al., 2015). Similarly, the expression of *TaREM4.1*, *TaREM4.2* and *TaREM4.3* increased in wheat, which improved tolerance in low-temperature stress (Badawi et al., 2019). Therefore, the remorin protein plays an important role in plant stress resistance.

Tomato (*Solanum lycopersicum* L.) is a fleshy fruit model plant and highly significant vegetable crops grown worldwide (Gerszberg et al., 2015). Different unfavorable environmental conditions significantly reduce the productivity and fruit quality of tomato (Wai et al., 2020). Remorin genes are ubiquitous in plants, playing key regulatory roles in many biological processes such as plant development and stress response. However, the *remorin* family genes in tomato are unknown, and no comprehensive analysis of this family in tomato has been reported. In this study, we identified the *remorin* gene family members and analyzed their structural characteristics, chromosomal distribution, phylogenetic relationship, collinearity, conserved motifs and promoter elements. To better understand the potential functions of the *remorin* gene family in tomato, we performed gene expression analysis in several tissues and in response to various stresses and hormone treatments. Our results provided a theoretical foundation for exploring the potential functions of *SlREM* genes in abiotic stress responses and regulating development.

## Materials and methods

### Plant materials and growth treatments

Seeds of wild-type (WT) tomato (*Solanum lycopersicum* Mill. cv. Ailsa Craig) were planted in potted soil, and seedlings were grown under normal temperature conditions (16 h light/8 h dark photoperiod). All hormone treatments seedlings were performed using comparable growth at 28 days of age, and then the tomato seedlings were sprayed with water, 100 μM ABA, 50 μM MeJA and 50 μM SA solutions (control), respectively. For NaCl treatment, the seedlings were treated with 200 mM NaCl solution. For low temperature treatment, the seedlings were placed in a growth cabinet at 4°C for 24 h. For drought treatment, the seedlings were transferred to 1/2 nutrient solution containing 20% PEG-6000. Meanwhile, the following samples were then collected: the tissues of roots, stems, leaves, shoot apexes, flower buds, full blooming flowers at the anthesis stage, and green and mature fruits. Tomato leaves were collected at 0, 6, 12 and 24 h after stress treatment. The collected samples were frozen in nitrogen and stored at −80°C until use. All samples were tested with three biological replicates, and each replicate consisted of ten seedlings.

### Identification of the *SlREM* genes in the tomato genome

All tomato genome sequence and protein sequences were downloaded from Sol Genomics Network. The HMM of the REM protein domain (remorin-C and remorin-N) was downloaded from the Pfam database (http://pfam.sanger.ac.uk/). HMMER 3.1 was used to screened protein sequences containing remorin domains from the genome database. The number of putative SlREM amino acids, molecular weight (MW), theoretical isoelectric point (pI) were analyzed using the ExPASy (website https://web.expasy.org/protparam/) (Xiong et al., 2015). The subcellular localization of the REM proteins was carried out with Wolf PSORT (https://wolfpsort.hgc.jp/) (Artimo et al., 2012).

### Chromosome localization and collinearity analysis

TBtools software was used to was drawn the chromosome distribution of *SlREMs* based on the tomato genome (Chen et al., 2020). MCScanX was adopted to analyze the collinearity of *REM* genes between *Arabidopsis* and tomato (Wang et al., 2012), and then the collinearity diagram was drawn with MapChart sotware (Voorrips, 2002). We used PAL2NAL (http://www.bork.embl.de/pal2nal/index.cgi)? to calculate the d_N_/d_S_ value of duplicate gene pairs (Goldman and Yang, 1994).

### Construction of phylogenetic tree

*Arabidopsi*s, rice and maize REM protein sequences were obtained from the Ensembl Plant database. We compared the identified SlREM sequences with the amino acid sequences of AtREM, OsREM and ZmREM proteins with MEGA7 program (Kumar et al., 2016). Subsequently, a multiple sequence alignment was used to construct a maximum likelihood tree with the LG + Gamma model and a bootstrap value of 1000 replicates in MEGA7.

### Analysis of gene exon-intron structures and protein conserved motifs

The structure of the *SlREMs* gene in tomato was drawn using the Gene Structure View tool of TBtools according to the introns-exon position information (Chen et al., 2020). The conserved domains of the tomato remorin proteins was identified by MEME website (http://meme-suite.org/tools/meme). The maximum number of motif number was set as 10, and the other parameters were set as default values (Bailey et al., 2009).

### Analysis of cis-acting elements in *SlREM* promoter regions

The 2000 bp sequences located upstream of the translation initiation codon for the *SlREM* genes was extracted using TBtools. The promoter elements in the sequences were analyzed with PlantCARE software (http://bioinformatics.psb.ugent.be/webtools/plantcare/html/) (Lescot et al., 2002).

### RNA extraction and quantitative real-time PCR

Total RNA was isolated from the collected samples using a Plant RNA Extraction kit (Tiangen, Beijing, China). The complementary DNA was synthesized using the StarScript II First-strand cDNA Synthesis Mix kit (GenStar, Beijing, China) with 2 μL RNA as the template. The tomato *EF1a* gene was used as an internal reference gene (Aoki et al., 2010). Then qRT-PCR was performed with a CFX96TM real-time fluorescent qPCR system (Bio-Rad, USA) using SYBR Green kit (Tiangen, Beijing, China). The gene-specific primers used for qRT-PCR were designed by Primer 6.0 in **Supplementary Table 1**. The amplification program conditions were as follows: step 1: 95°C for 2 min; step 2: 40 cycles of 95°C for 15 s, 60°C for 30 s; and step 3: melting curve analysis. Each sample was replicated three times. The relative expressions level of the *SlREM* genes was calculated using the 2^-ΔΔCT^ method (Livak and Schmittgen, 2001). SPSS 20.0 was used to analyze the relative expressions and Origin 9.0 was used to complete the histogram of relative expression.

## Results

### Genome-wide identification and sequence analysis of *SlREM* genes

In this study, a total of 17 putative *REM* genes were identified in the tomato genome according to the two domain, N-terminal (Pfam ID PF03766) and C-terminal (Pfam ID PF03763) regions of the encoded proteins and validating in SMART Conserved Domain Search Service. These 17 *REM* genes were sequentially renamed from *SlREM1* to *SlREM17* based on their physical location on the chromosome basic information. The physical and chemical properties of *SlREM* genes were analyzed (**Table 1**). The result showed the predicted SlREM protein sequences ranged from 153 amino acids (SlREM2) to 589 amino acids (SlREM9). The predicated molecular weight and isoelectric point (pI) ranged from 17.10 kDa (SlREM12)–64.96 kDa (SlREM9), and 5.64 (SlREM5)–10.13 (SlREM14). The putative SlREM protein subcellular localizations showed that most SlREM proteins might be located in the nucleus, which, two REMs proteins (SlREM14 and SlREM17) might be localized in the cytoplasm.

**Table 1:** Analysis of amino acid sequence characteristics of *SlREM* gene family in tomato.

Gene name	Gene ID	Chr^a^	AA^b^ (aa)	Mw^c^ (kDa)	pI^d^	SL^e^
SlREM1	Solyc01g008180.3	1	522	58.11	8.81	Nuclear
SlREM2	Solyc01g094370.3	1	174	19.17	9.03	Nuclear
SlREM3	Solyc02g064990.3	2	387	42.69	9.36	Nuclear
SlREM4	Solyc02g090900.3	2	377	41.79	9.78	Nuclear
SlREM5	Solyc03g025850.3	3	197	21.85	5.64	Nuclear
SlREM6	Solyc03g123590.3	3	373	41.52	6.65	Nuclear
SlREM7	Solyc04g005470.3	4	315	36.09	9.84	Nuclear
SlREM8	Solyc04g015520.3	4	303	34.18	6.57	Nuclear
SlREM9	Solyc04g078810.3	4	589	64.96	9.92	Nuclear
SlREM10	Solyc05g012550.3	5	385	42.98	9.76	Nuclear
SlREM11	Solyc05g014710.3	5	335	37.80	9.02	Nuclear
SlREM12	Solyc05g048820.3	5	153	17.10	9.74	Nuclear
SlREM13	Solyc06g035920.3.	6	185	20.52	7.67	Nuclear
SlREM14	Solyc06g069590.3	6	196	22.37	10.13	Cytosol
SlREM15	Solyc08g045640.3	8	494	56.29	9.61	Nuclear
SlREM16	Solyc10g017540.3	10	199	22.29	8.97	Nuclear
SlREM17	Solyc10g080220.2	10	738	82.80	8.23	Cytosol

^a^Chromosome location.

^b^Amino acid number.

^c^Molecular weight.

^d^Theoretical isoelectric point.

^e^Subcellular localization.

### Chromosomal location and collinearity analysis of the *SlREM* genes in tomato

**Figure 1:**
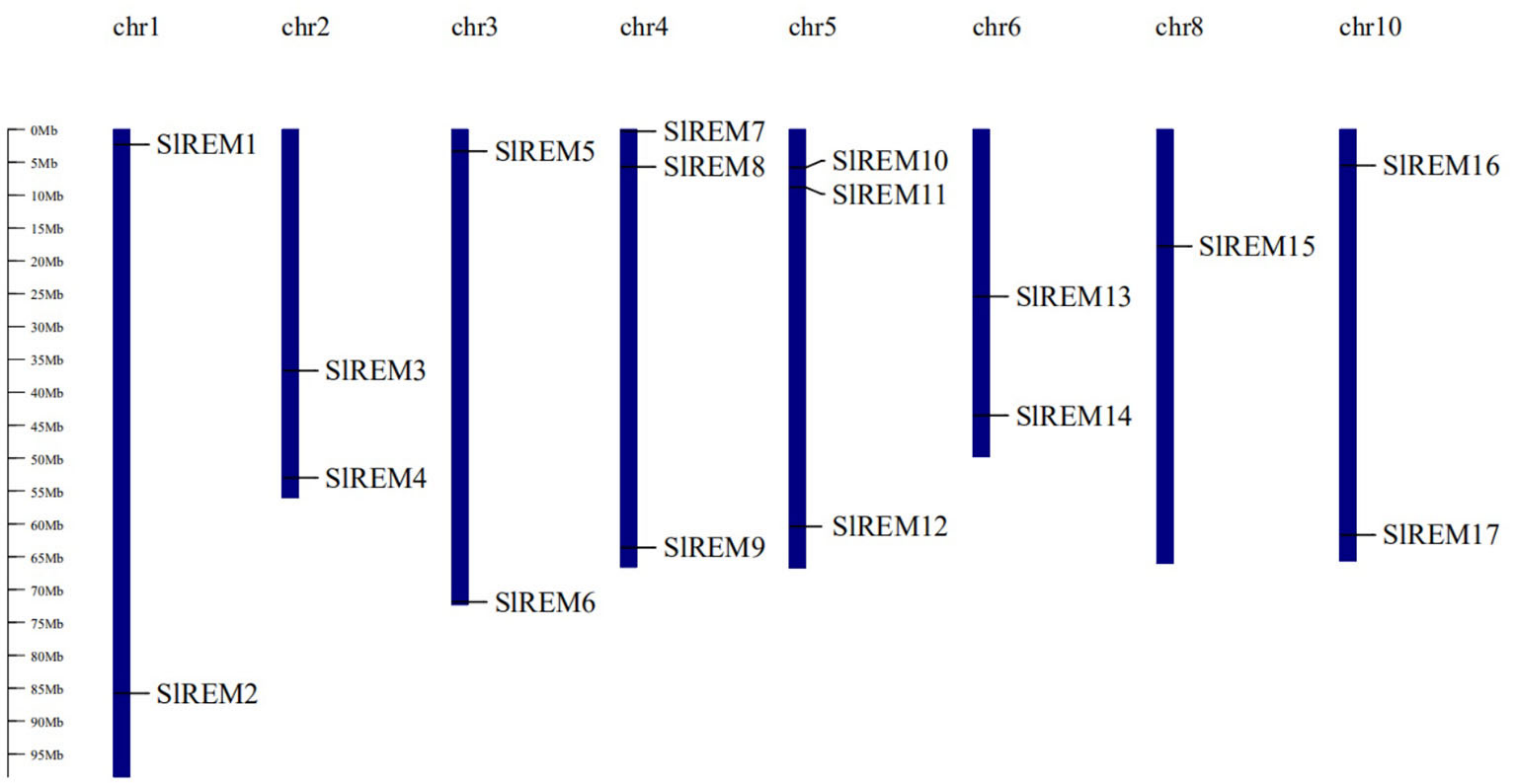
Chromosomal distribution and localization of *SlREM* family genes in tomato. The chromosome names are shown at the top of each chromosome, and chromosome numbers are listed above chromosomes. The chromosome scale is in millions of bases (Mb) on the left.

**Figure 2:**
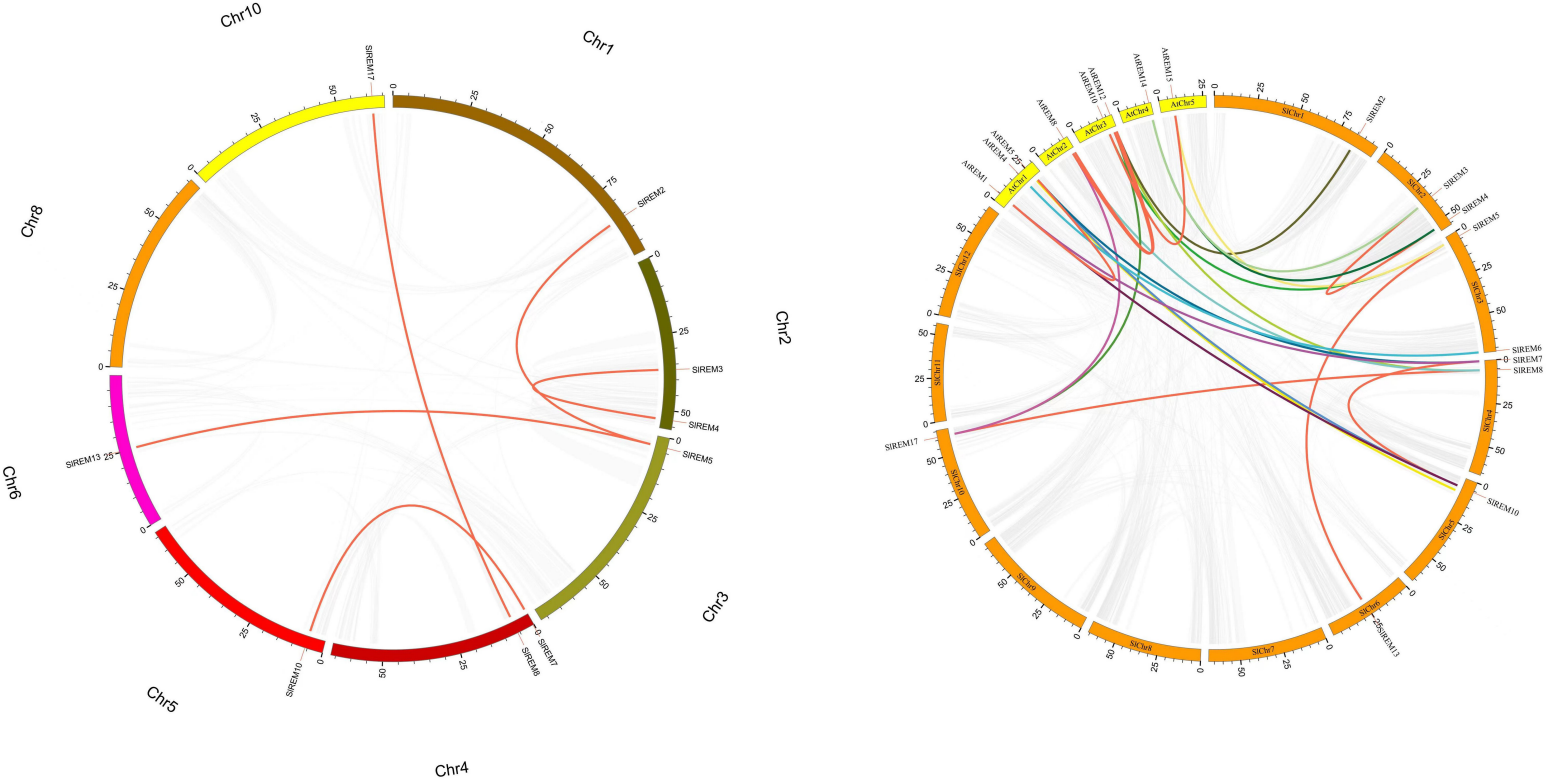
Collinear analysis of the *REM* gene family in tomato. a Chromosomes 1–12 are represented by different colors rectangles. The gray lines indicate synteny blocks in the tomato genome, while orange lines between chromosomes delineate segmental duplicated gene pairs. b Synteny analysis of *REMs* between tomato and *Arabidopsis*. Gray lines denote the collinear blocks between tomato and *Arabidopsis* genomes and the lines of different colors denote the syntenic gene pairs of *REMs*. Orange rectangles represent the tomato chromosomes (1–12) and yellow rectangles represent the *Arabidopsis* chromosomes (1–5).

The 17 of *SlREM* genes were distributed unevenly across the 8 chromosomes (Chr). Most *SlREM* genes were found on Chr 4 (*SlREM7*, *SlREM8* and *SlREM*9) and Chr 5 (*SlREM10*, *SlREM11* and *SlREM12*). Chr 8 had only one *SlREM* gene (*SlREM16*). Chr 7, 9 11 and 12 lacked *SlREM* genes (**Figure 1**). Closely related genes on the same chromosome that are less than 200 kb apart are defined as tandem duplication, otherwise they are defined as segmental duplication (Cheung et al., 2003). To better understand the expansion mechanism of the *SlREMs*, we examined the genome segmental and tandem duplication events in *SlREM* gene family. We found 5 segmental duplication events among the *SlREM* gene pairs (**Figure 2A**). In the five pairs of collinear relationships, *SlREM5* was paired with *SlREM2* and *SlREM13*, respectively, while the others were one to one paired.

Moreover, we also examined the *REM* homologous gene pairs between tomato and *Arabidopsis*. The results showed that there were 15 collinear gene pairs between 17 *SlREMs* and 16 *AtREMs* (**Figure 2B**; **Supplementary Table 2**). *SlREM5*, *SlREM7*, *SlREM8*, *SlREM10* and *SlREM17* were collinear with two *AtREM* genes (*AtREM10*–*AtREM15*, *AtREM6*–*AtREM1*, *AtREM11*–*AtREM8*, *AtREM6*–*AtREM1* and *AtREM11*–*AtREM8*), respectively. *SlREM2*, *SlREM3*, *SlREM4*, *SlREM6* and *SlREM11* were collinear with one *AtREM* gene, respectively.

In order to further survey the evolutionary constraints of *REM* gene family, the non-synonymous (d_N_), and synonymous (d_S_) substitution rates and d_N_/d_S_ values were evaluated for the segmentally duplicated gene pairs among tomato and *Arabidopsis* (**Supplementary Table 3**). Where a d_N_/d_S_ ratio >1 is the positive selection, a ratio equal to 1 is the neutral selection, and a ratio <1 is the purifying selection (Yadav et al., 2015). The d_N_/d_S_ value of all tomato gene pairs was less than 1, indicating that most of the SlREM genes were purifying selection. Most pairs of genes in tomato and *Arabidopsis* had d_N_/d_S_ less than 1, indicates that the *SlREM* gene family primarily underwent purifying selection. Five pairs of genes in tomato and *Arabidopsis* had d_N_/d_S_ greater than 1, indicative of positive selection. The one gene pairs with d_N_/d_S_ value close to 1, indicative of neutral selection.

### Phylogenetic analysis in tomato REM proteins

**Figure 3:**
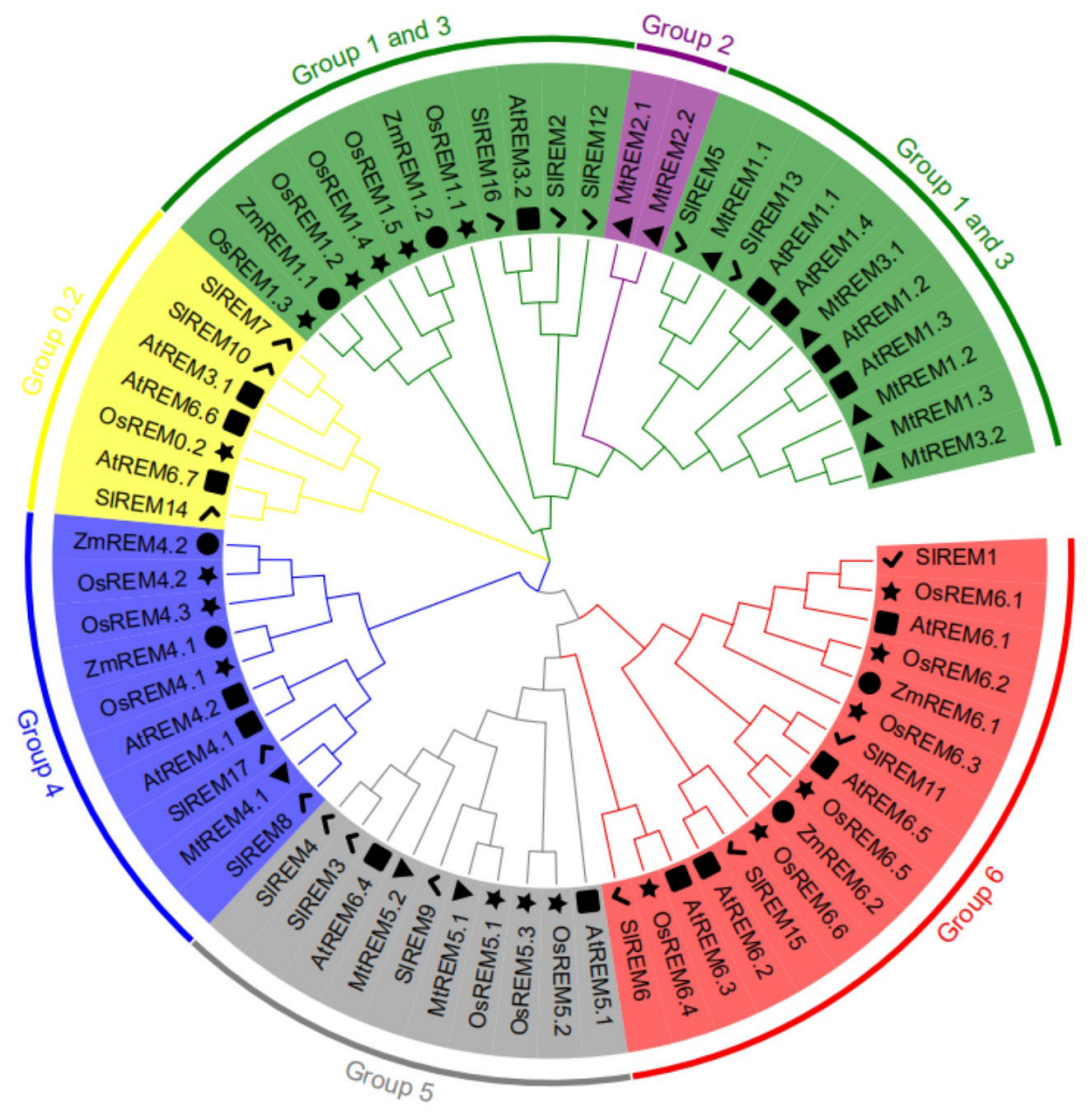
Phylogenetic relationship of SlREM proteins among *Arabidopsis* (16), *M. truncatula* (10), rice (18), maize (6) and tomato (17) using the Maximum Likelihood Method (1000 bootstrap). The lines of different colors indicate different groups. Square, triangles, star, circles and check mark represent remorin proteins of Arabidopsis, M. truncatula, rice, maize and tomato, respectively.

**Figure 4:**
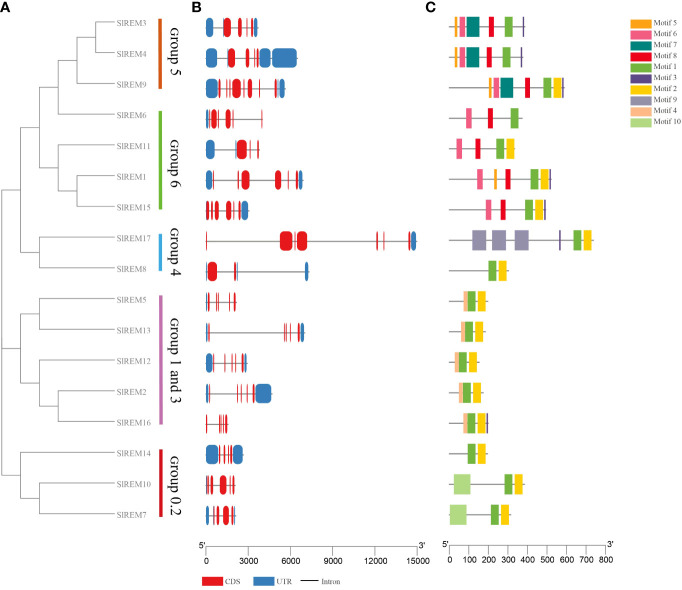
Phylogenetic tree, gene structure and conserved motif analysis of *SlREMs*. **(A)** The phylogenetic tree of *SlREM* is divided into five groups. **(B)** Exon–intron structures of *SlREM* genes. **(C)** Distribution of all motifs identified by MEME. Different colors of boxes represent different motifs in the corresponding positions of each protein.

To analyze the phylogenetic relationships among remorins, total 67 REM proteins were collected from *Arabidopsis* (16), *M. truncatula* (10), rice (18), maize (10) and tomato (17) to construct the phylogenetic tree (**Figure 3**; **Supplementary Table 4**). These REM proteins were clustered in six main evolutionary branches. Among the 67 REM proteins, 22 belonged to groups 1–3, 7 belonged to group 0.2, 10 belonged to group 4, 10 belonged to group 5, and 16 belonged to group 6. Group 2 comprised only two proteins from *M. truncatula*. The 17 SlREM proteins from tomato were divided into five groups on the basis of their structural features and phylogenetic relationships (**Figure 4A**; **Supplementary Table 4**). Five SlREM proteins (SlREM2, 5, 12, 13 and 16) belonged to groups 1–3. The N-terminal and C-terminal remorin domains were all detected in SlREM2, 5, 12, 13 and 16, which belonged to group 1. Three SlREM belonged to group 0.2, two SlREM proteins belonged to group 4, three belonged to group 5, and four belonged to group 6.

### Gene structure and conserved motif analysis of *SlREM genes*

The exon/intron patterns and conserved motifs based on their phylogenetic relations (**Figure 4A**). The results showed that the number of exons varied from 2 to 8 in *SlREM* genes (**Figure 4B**), with two exon in *SlREM8*, three in *SlREM11*, four in *SlREM3*, *4*, 7, 14 and *16*, five in *SlREM2, 5, 10, 12* and *13*, six in *SlREM1*, *6* and *15*, seven in *SlREM17* and eight in *SlREM9*.

A total of 10 conserved motifs among different groups of SlREM proteins were identified using MEME tool. The lengths of these motifs range from 8 to 86 amino acids (**Figure 4C**; **Supplementary Figure 1**; **Supplementary Table 5**). The SlREM protein family members have highly conserved motifs, and all SlREMs contain motifs 1. Motifs 1, 2, and 3, which were detected in the remorin C-terminal domain, were widely present in all the SlREM proteins. Motif 4, which was identified in the N-terminal region, was present in SlREM2, 5, 12, 13 and 16. In addition, the SlREM proteins in the same group shared common motifs distribution patterns.

### Cis-element analysis of the *SlREM*s promoter regions in tomato

**Figure 5:**
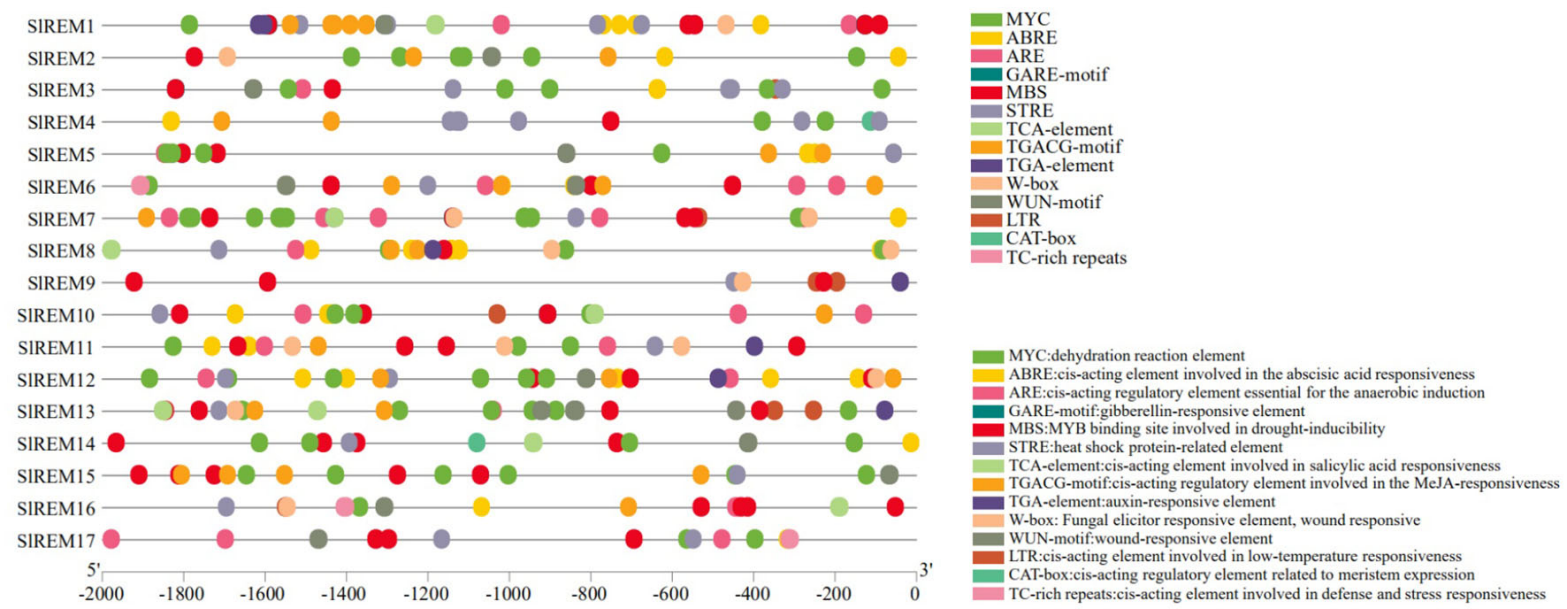
Putative cis-elements distributions of *SlREM* family gene promoters. The homeopathic elements represented by different color boxes and their names and functions.

To elucidate the expression and regulatory mechanism of 17 *SlREM* gene family members in tomato, we analyzed the promoter sequence of *SlREM* genes using the PlantCARE tool (**Figure 5**; **Supplementary Figure 2**). The 13 predicted hormone–related cis-elements, such as the abscisic acid responsive element (ABRE), gibberellic acid (GARE-motif), salicylic acid (SA) (TCA-element) responses, methyl jasmonate (MeJA) responsive element; TGACG-motif), and auxin (AuxR-core, TGA-box, and TGA-element). In addition, stress-related cis-elements were abundant in the promoter regions of *SlREM* genes, including dehydration reaction element (MYC); the anaerobic induction element (ARE); drought-responsive element (MBS); heat shock protein responsive element (STRE); pathogen response element (W-box); trauma response element (WUN-motif); low-temperature responsiveness (LTR) and defense-and stress-responsive elements (TC-rich repeats). In addition, meristematic expression regulation elements (CAT-box) was present in individual *SlREM* promoters. The different types and numbers of cis-elements in the promoters may be responsible for multiple functions of *SlREMs* through complex regulatory mechanisms.

### Expression analysis of tomato *SlREM* genes in different tissues

**Figure 6:**
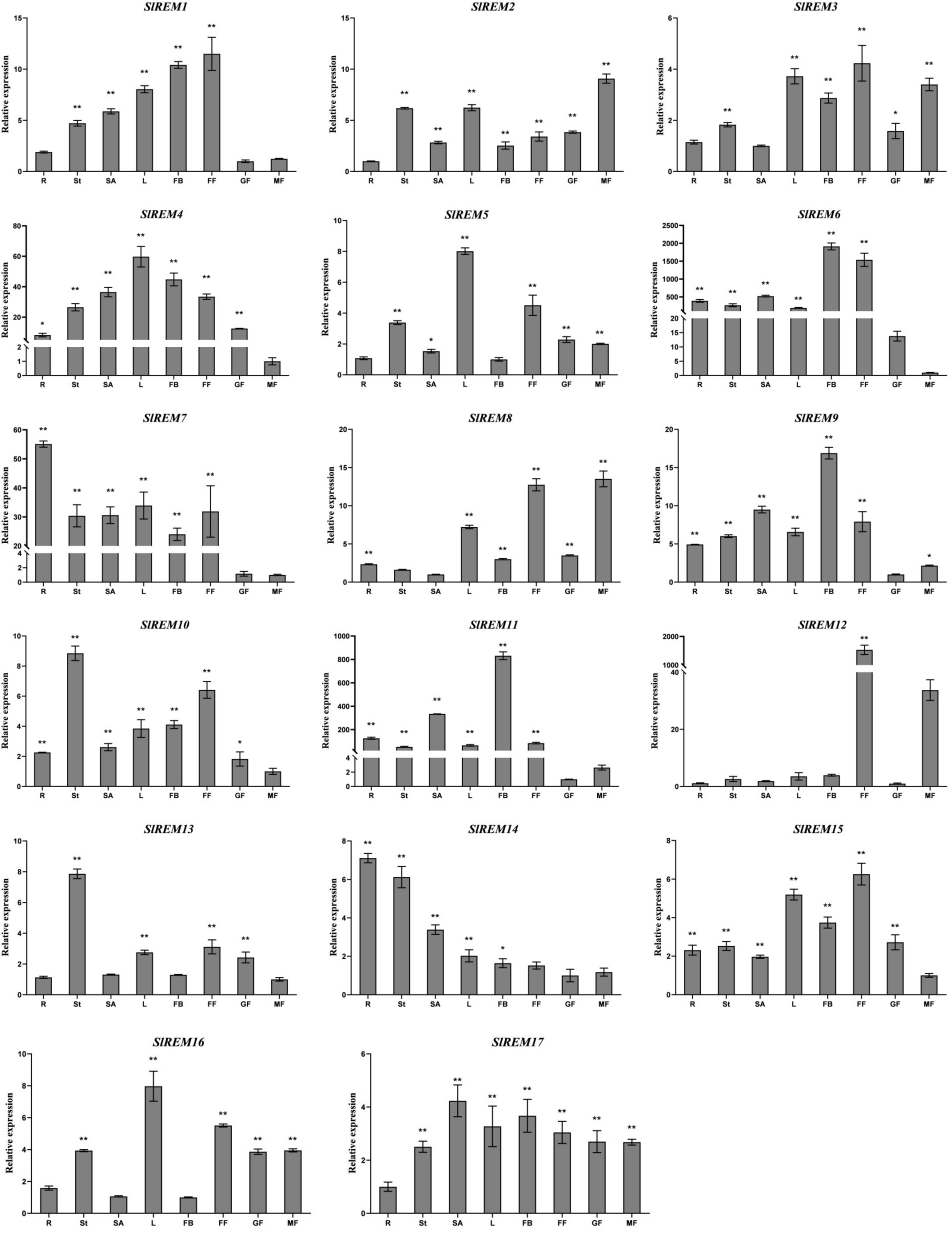
Expression levels of *SlREMs* in various tomato tissues. Tissue specific of *SlREM* expression was tested by qRT-PCR. R, St, L, SA, FB, FF, GF and MG represent root, stem, young leaves, shoot apexes, flower bud, full blooming flower, green fruit and mature fruit, respectively. Error bars represent the standard error (SE) of three biological replicates. The *p* value was calculated through student’s *t*-test. Asterisk indicate the significant difference compared with control. * and ** indicate p<0.05 and p<0.01, respectively.

To examine the possible functions of *SlREM* genes in the growth and development of tomato, the expression patterns of *SlREM* genes in different tissues and organs were analyzed using qRT-PCR (**Figure 6**). The *SlREM* genes were expressed in diverse tissues. The *SlREM* genes were expressed in different tissues, indicating they have diverse functions. Among the 17 *SlREM* genes, *SlREM2*, *4*, *9*, *10*, *15* and *17* were highly expressed in all tissues. *SlREM13* were highly expressed in the stem, leaves, flower and green fruit. The *SlREM14* expression levels were also high in all tissues, except for in the flower and mature fruit. The *SlREM16* expression levels were highly abundant in all tissues, except for in the root and flower bud. The *SlREM1* expression levels were also high in all tissues, except for in the root and mature fruit. Moreover, *SlREM6* and *SlREM7* was not expressed in green fruit, *SlREM11* was not not expressed in mature fruit, *SlREM8* was not expressed in the stem, *SlREM5* was not expressed in the flower bud, *SlREM3* was not expressed in the root. *SlREM12* was specifically expressed in the flower. The results indicated that these *SlREM* genes might play a key role in tomato growth and development.

### Expression patterns of *SlREM* genes under different treatments

**Figure 7:**
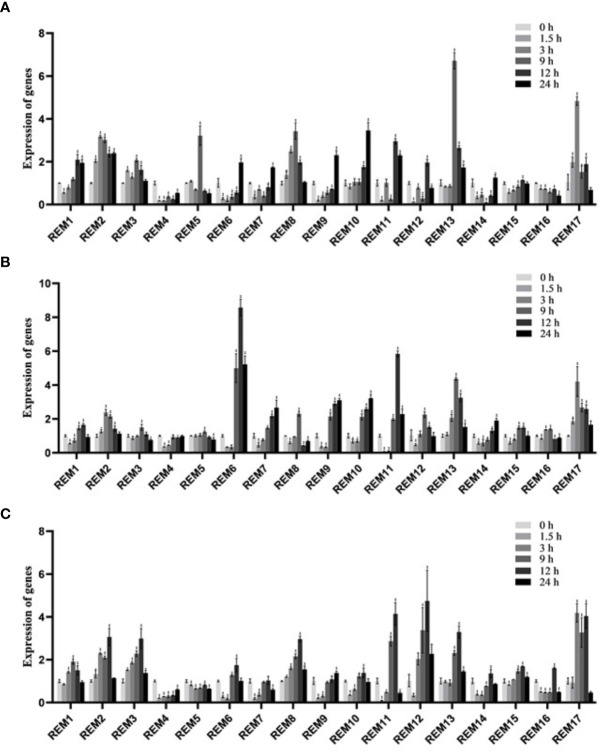
Expressions analysis of *SlREMs* genes in response to ABA **(A)**, MeJA **(B)**, SA **(C)** treatments. The expression levels of *SlREMs* were tested by qRT-PCR. 0, 6, 12 and 24 h represent 0, 6, 12 and 24 h under different treatment, respectively. Error bars represent the standard error (SE) of three biological replicates. The *p* value was calculated through student’s *t*-test. Asterisk indicate the significant difference compared with control. * and ** indicate p<0.05 and p<0.01, respectively.

For explore the potential responsiveness of *SlREM* genes to hormones, qRT-PCR experiments were carried out under different hormones treatments (**Figure 7**). Under ABA treatment (**Figure 7A**), all of *SlREMs* had significantly differences compared with 0 h. Compared to the 0 h, the expressions of *SlREM2*, *SlRE*M10 and *SlREM* 13 were up-regulated, and the expressions of *SlREM4* and *SlREM16* were down-regulated. The expression of *SlREM2* and *SlREM8* were increased firstly and then decreased. *SlREM6* and *SlREM9* showed early down-regulation followed by up-regulation. Notably, *SlREM13* showed the most significant response to ABA, and its expression increased more than 6 times at 9 h compared with the 0 h. Under 50 MeJA treatment (**Figure 7B**), *SlREM2*, *SlREM6*, *SlREM13* and *SlREM17* were significantly up-regulated (**Figure 7C**), and the expressions of *SlREM4* was down-regulated. Notably, *SlREM12* showed significantly regulated by SA treatment, and its expression increased more than 4 times at 12 h compared with the 0 h.

**Figure 8:**
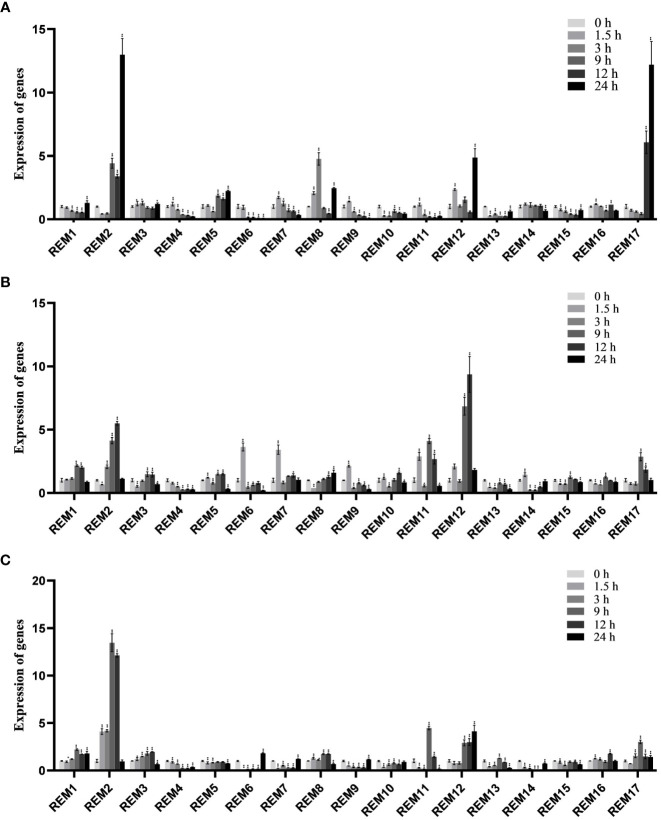
Expressions analysis of *SlREMs* genes in response to cold **(A)**, drought **(B)** and NaCl **(C)** treatments. The expression levels of *SlREMs* were tested by qRT-PCR. 0, 1.5, 3, 9, 12 and 24 h represent 0, 1.5, 3, 9, 12 and 24 h under different treatment, respectively. Error bars represent the standard error (SE) of three biological replicates. The *p* value was calculated through student’s *t*-test. Asterisk indicate the significant difference compared with control. * and ** indicate p<0.05 and p<0.01, respectively.

To analyze the expression pattern of tomato *SlREM* members to abiotic stresses, the expression profile under abiotic stresses (cold, drought and NaCl) was analyzed using qRT-PCR (**Figure 8**). Under low-temperature treatment (**Figure 8A**), *SlREM2* and *SlREM17* were significantly up-regulated (>10-fold), while expression levels peaked at 24 h. Notably, *SlREM12* were up-regulated after drought treatment, and its expression increased more than 9 times at 12 h compared with the 0 h (**Figure 8B**). Under NaCl treatment (**Figure 8C**), *SlREM1* and *SlREM12* were up-regulated. The expression of *SlREM2* showed early responses to NaCl stress, though their expression decreased at 9 h. Our results provide a basis for the the potential important functions of the *SlREM* genes in the future.

## Discussion

*Remorin* family genes are involved in developmental processes and abiotic stress responses (Checker and Khurana, 2013; Li et al., 2014; Yue et al., 2014; Gui et al., 2016; Cai et al., 2018; Badawi et al., 2019). REM gene families have been identified in several higher plants, such as *Arabidopsis* (Bhat et al., 2005), rice (Raffaele et al., 2007), wheat (*Triticum aestivum* L.) (Badawi et al., 2019), foxtail millet (*Setaria italica*) (Wang et al., 2022), potato (*Solanum tuberosum* L.) (Jacinto et al., 1993), tobacco (*Nicotiana tabacum* L.) (Mongrand et al., 2004) and *Medicago trunculata* Gaertn. (Lefebvre et al., 2007). However, the REM gene family in tomato has not been studied in detail. In this study, we comprehensively analyzed *SlREM* genes in tomato, including genome-wide identification, physicochemical properties, chromosome location, collinear relationship, gene structure and conserved motifs, cis-acting elements and expression patterns. Seventeen *SlREM* genes were identified in tomato genome and renamed as *SlREM1-17* based on their chromosomal location (**Table 1**). Compared with rice (20), wheat (20) and foxtail millet (21), the amount of REM in tomato was much less, which might be related to the genome sizes and genome-wide doubling events.

Chromosome mapping (**Figure 1**) found that the *SlREM* genes located on Chr 4 and 5 were the most distributed, with the number of 3 and 3, respectively. Chr 8 had only one *SlREM* gene (*SlREM16*). Chr 7, 9 11 and 12 lacked *SlREM* genes. Tandem and segmental duplications have played important roles in expanding the gene families in the plant genome (Cannon et al., 2004; Flagel and Wendel, 2009), and segmental duplication is more conducive to the maintenance of gene function in the process of gene replication (Lynch and Conery, 2000). In our study, the *SlREM* gene had no tandem duplication gene pairs, but that there were 5 homologous gene pairs existed among *SlREMs* and 15 pairs of collinear genes in *Arabidopsis* and tomato (**Figures 2A, B** **)**, indicating that *SlREM* genes expand primarily through segmental duplication of the chromosome. In addition, the amplification of *OsREM* genes in rice is also through fragment replication, which was consistent with our results. Furthermore, the d_N_/d_S_ values of most *SlREM* duplication gene pairs was less than 1, indicating that most *SlREM* genes underwent purification selection. The calculated d_N_/d_S_ value of five *SlREM* duplication gene pairswas was greater than 1, indicating positive selection pressures. The one gene pairs with d_N_/d_S_ value close to 1 was likely affected by neutral evolutionary processes. This analysis indicates that purification selection plays a critical role in the evolution of the *SlREM* gene family and could help to maintain the their the basic function of genes (**Supplementary Table 3**).

According to the phylogenetic tree, SlREM proteins were clustered into five groups (**Figure 3**). And they have a close evolutionary relationship with SlREM proteins in dicot *Arabidopsis*. This suggests that SlREMs are highly conserved in plant evolution. In addition, we analyzed the exon-intron arrangements and conserved motifs of *SlREM* genes based on their phylogenetic relations (**Figure 4A**). The exon/intron structure of genes is an important indicator of the evolutionary relationship among the members of a gene family (Long et al., 2003). Our results showed that the number of exons in *SlREM* genes were various, ranging from 2 to 8, and tomato *REM* genes in the same group had similar exon/intron structures (**Figure 4B**). While the distribution pattern of exon/intron structures was different from genes in the same group, which may be due to a number of reasons (Rogozin et al., 2005). A total of 10 conserved motifs were identified in the amino acid sequence of *SlREM* genes. Additionally, the SlREM proteins in the same group shared similar motifs distribution patterns (**Figure 4C**), indicating that SlREM proteins within the same group have conserved functions. In groups 1-3, the SlREM proteins primarily comprised motifs 1, 2, 3, and 4, which are the basic remorin motifs. Motifs 1, 2, and 3, which were detected in the remorin C-terminal domain, were widely present in all the SlREM proteins, suggesting that they have been conserved during evolution, thus maintaining the essential characteristics of the remorin family. In contrast, motif 4, which was identified in the N-terminal region, has been reported that it varies (Bariola et al., 2004). Motifs 5, 6, 7, and 8 were detected in the remorin proteins of group 5, whereas motifs 5, 6 and 8 were the characteristic domains in group 6. The motifs in group 4 were relatively complex. Motifs 9 was detected in the remorin proteins of group 4, and motifs 10 was detected in the remorin proteins of group 0.2 (**Figure 4C**; **Supplementary Figure 1**; **Supplementary Table 5**). Moreover, the diversity of motifs indicates functional differences among the examined proteins.

The cis-acting elements in the gene promoter region are binding sites for specific transcription factors, which regulate the precise initiation and transcription efficiency of gene transcription (Carrier et al., 2020). So far, many cis-acting elements have been well characterized and grouped into different groups (Hadiarto and Tran, 2011). We have identified a number of cis-acting elements in the promoter regions of tomato REM genes, including stress-responsive regulatory elements: MYC, STRE, LTR, ARE, MBS, WUN-motif and TC-rich repeats; hormone-responsive regulatory elements: ABRE, GARE motif, TCA-element, TGACG-motif, and TGA-element (**Figure 5**; **Supplementary Figure 2**). The widely present of these cis-acting regulatory elements of SlREM suggested that they might play a crucial role in plant growth, development and stress resistance.

The specific expression patterns of genes at different developmental stages in different tissues provide a new insights into the function of tomato *REM* family genes. In plants, *REM* genes have been identified in many tissues. In foxtail millet and A. thaliana, several members of the REM family were expressed in the vascular system (Bariola et al., 2004; Yue et al., 2014). In *P. deltoides*, *PdREM* is mainly expressed in leaf buds, and immature and mature phloem (Li et al., 2013). In wheat, *TaREM* was expressed in different tissues like leaves, stems, crowns, and roots (Badawi et al., 2019). In the present study, qRT-PCR expression analysis of *SlREMs* showed that most of the examined tomato remorin genes were expressed in all or some of the eight analyzed tissues. Among them, *SlREM1*, *SlREM3*, *SlREM12* and *SlREM15* were highly expressed in flower. Thus, *SlREM* members may function in flower and fruit development. Simultaneously, we also detected a abundant expression of tomato *SlREM* genes in the mature fruit tissues, suggesting a different regulation mechanism of *SlREMs* in fruit ripening (**Figure 6**). In summary, these results show that the *SlREM* genes might play important roles in tomato growth and development.

Plant hormones, such as ABA, SA and MeJA, regulate plant responses to biotic and biotic stresses (Jones, 2016; Kumar, 2014; Carvalhais et al., 2017). In *A. thaliana*, *REMs* are induced by binding of transcription factors to specific cis-elements, including ABA-dependent and ABA-independent pathways (Raffaele et al., 2007). In addition to ABA, *REM* genes are regulated by several hormones including SA, MeJA, and brassinosteroids (Gui et al., 2016; Kong et al., 2016; Hu et al., 2013). In this study, the expression levels of most *SlREM* genes were differentially regulated under the ABA, SA and MeJA treatments. For example, the expressions of *SlREM2*, *SlRE*M10 and *SlREM* 13 were up-regulated under ABA treatment; *SlREM2*, *SlREM6*, *SlREM13*, and *SlREM17* were significantly up-regulated under MeJA treatment; the expressions of *SlREM3*, *SlREM8*, *SlREM12, SlREM13* and *SlREM17* were up-regulated under SA treatment (**Figure 7**). Our results indicated that *SlREM* genes might be participate in the regulation of ABA, JA and SA signaling pathways. In addition, *REM* genes are also involved in responds to different abiotic stresses. Overexpression of *MiREM* gene in *Arabidopsis* improved drought and salt-stress tolerance (Checker and Khurana, 2013). The expression of *SiREM6* increased after high-salt, low-temperature and exogenous ABA conditions in foxtail millet (*Setaria italica*) (Li et al., 2013). Overexpression of *SiREM6* increased the tolerance of *Arabidopsis* to high salt during germination and seedling stages (Yue et al., 2014). *DaCBF7* from *Deschampsia antarctica* in transgenic rice plants upregulates *remorin* gene expression, thus improving cold tolerance (Byun et al., 2015). In our study, we detected the expression levels of 17 *SlREM* genes under low-temperature, drought, salt stresses. We found that most *SlREM* genes showed differentially regulated in response to different abiotic stresses (**Figure 8**). These results indicate that *SlREM* genes may play an important role in the response to abiotic stress in tomato resistance and their functions need to be further explored.

## Conclusions

In this study, a total of 17 REM motif-containing genes in tomato genome and performed a systematically study on *SlREM* genes. This included physicochemical properties, chromosome distribution, collinearity analysis, phylogenetic relationships, gene structures, conserved motifs, promoter cis-acting elements and expression profiles. The 17 *SlREM* genes were divided into 6 groups based on their protein sequences and unevenly distributed across the on eight chromosomes. The *SlREM* gene structures and motif compositions were similar. Promoter sequence analysis indicated that there were some tissue-specific and stress-related elements in *SlREMs* promoter regions. Expression analysis of *SlREM* family genes indicated that *SlREM* genes plays an important role in tomato growth and development, stress and hormone responses. In summary, the present work provides a foundation for future study on the role of *SlREM* gene functions in tomato.

## Data availability statement

The original contributions presented in the study are included in the article/**Supplementary Materials**. Further inquiries can be directed to the corresponding authors.

## Author contributions

HL, XW, HM, and MZ designed the experiment. HL, XW and YZ performed the experiment. HL, XW, SC, and JL performed the data analysis. HL and XW wrote the manuscript. HM and MZ paid for part of the study and provided revised suggestions. All authors contributed to the article and approved the submitted version.

## References

[B1] Aoki K. Yano K. Suzuki A. Kawamura S. Sakurai N. Suda K.. (2010). Large-Scale analysis of full-length cdnas from the tomato (*Solanum lycopersicum*) cultivar micro-tom, a reference system for the solanaceae genomics. BMC Genomics 11 (1), 210. doi: 10.1186/1471-2164-11-210 20350329 PMC2859864

[B2] Artimo P. Jonnalagedda M. Arnold K. Baratin D. Csardi G. de Castro E.. (2012). ExPASy: SIB bioinformatics resource portal. Nucleic Acids Res. 40 (W1), W597–W603. doi: 10.1093/nar/gks400 22661580 PMC3394269

[B3] Badawi M. A. Agharbaoui Z. Zayed M. Li Q. Byrns B. Zou J.. (2019). Genome-wide identification and characterization of the wheat remorin (TaREM) family during cold acclimation. Plant Genome. 12 (2) 180040. doi: 10.3835/plantgenome2018.06.0040 PMC12810125 31290927

[B4] Bailey T. L. Mikael B. Buske F. A. Martin F. Grant C. E. Luca C.. (2009). MEME SUITE: tools for motif discovery and searching. Nucleic Acids Res. 37, W202–W208. doi: 10.1093/nar/gkp335 19458158 PMC2703892

[B5] Bariola P. A. Retelska D. Stasiak A. Kammerer R. A. Fleming A. Hijri M.. (2004). Remorins form a novel family of coiled coil-forming oligomeric and filamentous proteins associated with apical, vascular and embryonic tissues in plants. Plant Mol. Biol. 55 (4), 579–594. doi: 10.1007/s11103-004-1520-4 15604702

[B6] Bhat R. A. Miklis M. Schmelzer E. Schulze-Lefert P. Panstruga R. (2005). Recruitment and interaction dynamics of plant penetration resistance components in a plasma membrane microdomain. P Natl. Acad. Sci. U.S.A. 102 (8), 3135–3140. doi: 10.1073/pnas.0500012102 PMC549507 15703292

[B7] Bray E. A. (2002). Abscisic acid regulation of gene expression during water-defificit stress in the era of the *Arabidopsis* genome. Plant Cell Environ. 25 (2), 153–161. doi: 10.1046/j.1365-3040.2002.00746.x 11841660

[B8] Byun M. Y. Lee J. Cui L. H. Kang Y. Oh T. K. Park H.. (2015). Constitutive expression of DaCBF7, an Antarctic vascular plant deschampsia antarctica CBF homolog, resulted in improved cold tolerance in transgenic rice plants. Plant Sci. 236, 61–74. doi: 10.1016/j.plantsci 26025521

[B9] Cai J. Qin G. Chen T. Tian S. (2018). The mode of action of remorin1 in regulating fruit ripening at transcriptional and post-transcriptional levels. New Phytol. 219 (4), 1406–1420. doi: 10.1111/nph.15264 29978907

[B10] Cannon S. B. Mitra A. Baumgarten A. Young N. D. May G. (2004). The roles of segmental and tandem gene duplication in the evolution of large gene families in *Arabidopsis thaliana* . BMC Plant Biol. 4, 10. doi: 10.1186/1471-2229-4-10 15171794 PMC446195

[B11] Carrier M. C. Ng Kwan Lim E. Jeannotte G. Massé E. (2020). Trans-acting effectors versus RNA cis-elements: a tightly knit regulatory mesh. Front. Microbiol. 11. doi: 10.3389/fmicb.2020.609237 PMC7769764 33384678

[B12] Carvalhais L. C. Schenk P. M. Dennis P. G. (2017). Jasmonic acid signalling and the plant holobiont. Curr. Opin. Microbiol. 37, 42–47. doi: 10.1016/j.mib.2017.03.009 28437665

[B13] Checker V. G. Khurana P. (2013). Molecular and functional characterization of mulberry EST encoding remorin (MiREM) involved in abiotic stress. Plant Cell Rep. 32 (11), 1729–1741. doi: 10.1007/s00299-013-1483-5 23942844

[B14] Chen C. Chen H. Zhang Y. Thomas H. R. Frank M. H. He Y.. (2020). TBtools: an integrative toolkit developed for interactive analyses of big biological data. Mol. Plant 13 (8), 1194–1202. doi: 10.1016/j.molp.2020.06.009 32585190

[B15] Cheung J. Estivill X. Khaja R. MacDonald J. R. Lau K. Tsui L. C.. (2003). Genome-wide detection of segmental duplications and potential assembly errors in the human genome sequence. Genome Biol. 4 (4), R25. doi: 10.1186/gb-2003-4-4-r25 12702206 PMC154576

[B16] Flagel L. E. Wendel J. F. (2009). Gene duplication and evolutionary novelty in plants. New Phytol. 183 (3), 557–564. doi: 10.1111/j.1469-8137.2009.02923.x 19555435

[B17] Gerszberg A. Hnatuszko-Konka K. Kowalczyk T. Kononowicz A. K. (2015). Tomato (*Solanum lycopersicum* l.) in the service of biotechnology. Plant Cell Tiss Org Culture. 120, 881–902. doi: 10.1007/s11240-014-0664-4

[B18] Goldman N. Yang Z. (1994). A codon-based model of nucleotide substitution for protein-coding DNA sequences. Mole Biol. Evol. 11 (5), 725–736. doi: 10.1093/oxfordjournals.molbev.a040153 7968486

[B19] Gong Z. Xiong L. Shi H. Yang S. Zhu J. K. (2020). Plant abiotic stress response and nutrient use efficiency. Sci. China Life Sci. 63 (5), 635–674. doi: 10.1007/s11427-020-1683-x 32246404

[B20] Gui J. Liu C. Shen J. Li L. (2014). Grain setting defect1, encoding a remorin protein, affects the grain setting in rice through regulating plasmodesmatal conductance. Plant Physiol. 166 (3), 1463–1478. doi: 10.1104/pp.114.246769 25253885 PMC4226345

[B21] Gui J. Zheng S. Liu C. Shen J. Li J. Li L. (2016). OsREM4.1 interacts with OsSERK1 to coordinate the interlinking between abscisic acid and brassinosteroid signaling in rice. Dev. Cell. 38 (2), 201–213. doi: 10.1016/j.devcel.2016.06.011 27424498

[B22] Gui J. Zheng S. Shen J. Li L. (2015). Grain setting defect1 (GSD1) function in rice depends on s-acylation and interacts with actin 1 (OsACT1) at its c-terminal. Front. Plant Sci. 6 doi: 10.3389/fpls.2015.00804 PMC4590517 26483819

[B23] Hadiarto T. Tran L. S. (2011). Progress studies of drought-responsive genes in rice. Plant Cell Rep. 30 (3), 297–310. doi: 10.1007/s00299-010-0956-z 21132431

[B24] Hu Y. Jiang L. Wang F. Yu D. (2013). Jasmonate regulates the inducer of cbf expression-C-repeat binding factor/DRE binding factor1 cascade and freezing tolerance in Arabidopsis. Plant Cell. 25(8), 2907–2924. doi: 10.1105/tpc.113.112631 23933884 PMC3784588

[B25] Jacinto T. Farmer E. E. Ryan C. A. (1993). Purification of potato leaf plasma membrane protein pp34, a protein phosphorylated in response to oligogalacturonide signals for defense and development. Plant Physiol. 103 (4), 1393–1397. doi: 10.1104/pp.103.4.1393 12232033 PMC159131

[B26] Jones A. M. (2016). A new look at stress: abscisic acid patterns and dynamics at high-resolution. New Phytol. 210 (1), 38–44. doi: 10.1111/nph.13552 26201893

[B27] Kong C. Luo Y. Duan T. Xue Z. Gao X. Zhao X.. (2016). Potato remorin gene StREMa4 cloning and its spatiotemporal expression pattern under Ralstonia solanacearum and plant hormones treatment. Phytoparasitica. 44(4), 575–584. doi: 10.1007/s12600-016-0536-z

[B28] Kumar D. (2014). Salicylic acid signaling in disease resistance. Plant Sci. 228, 127–134. doi: 10.1016/j.plantsci.2014.04.014 25438793

[B29] Kumar S. Stecher G. Tamura K. (2016). MEGA7: molecular evolutionary genetics analysis version 7.0 for bigger datasets. Mol. Biol. Evol. 33 (7), 1870–1874. doi: 10.1093/molbev/msw054 27004904 PMC8210823

[B30] Lefebvre B. Furt F. Hartmann M. A. Michaelson L. V. Carde J. P. Sargueil-Boiron F.. (2007). Characterization of lipid rafts from medicago truncatula root plasma membranes: a proteomic study reveals the presence of a raft-associated redox system. Plant Physiol. 144 (1), 402–418. doi: 10.1104/pp.106.094102 17337521 PMC1913791

[B31] Lescot M. Déhais P. Thijs G. Marchal K. Moreau Y. Van de Peer Y.. (2002). PlantCARE, a database of plant cis-acting regulatory elements and a portal to tools for in silico analysis of promoter sequences. Nucleic Acids Res. 30 (1), 325–327. doi: 10.1093/nar/30.1.325 11752327 PMC99092

[B32] Li S. Su X. Zhang B. Huang Q. Hu Z. Lu M. (2013). Molecular cloning and functional analysis of the populus deltoides remorin gene PdREM. Tree Physiol. 33 (10), 1111–1121. doi: 10.1093/treephys/tpt072 24072517

[B33] Li C. Yue J. Wu X. Xu C. Yu J. (2014). An ABA-responsive DRE-binding protein gene from setaria italica, SiARDP, the target gene of SiAREB, plays a critical role under drought stress. J. Exp. Bot. 65 (18), 5415–5427. doi: 10.1093/jxb/eru302 25071221 PMC4157718

[B34] Liu E. Liu Y. Wu G. Zeng S. Tran Thi T. G. Liang L.. (2016). Identification of a candidate gene for panicle length in rice (*Oryza sativa* l.) *Via* association and linkage analysis. Front. Plant Sci. 7. doi: 10.3389/fpls.2016.00596 PMC4853638 27200064

[B35] Livak K. J. Schmittgen T. D. (2001). Analysis of relative gene expression data using real-time quantitative PCR and the 2(-delta delta C(T)) method. Methods. 25 (4), 402–408. doi: 10.1006/meth.2001.1262 11846609

[B36] Long M. Betrán E. Thornton K. Wang W. (2003). The origin of new genes: glimpses from the young and old. Nat. Rev. Genet. 4 (11), 865–875. doi: 10.1038/nrg1204 14634634

[B37] Lynch M. Conery J. S. (2000). The evolutionary fate and consequences of duplicate genes. Science. 290 (5494), 1151–1155. doi: 10.1126/science.290.5494.1151 11073452

[B38] Marín M. Ott T. (2012). Phosphorylation of intrinsically disordered regions in remorin proteins. Front. Plant Sci. 3. doi: 10.3389/fpls.2012.00086 PMC3355724 22639670

[B39] Mongrand S. Morel J. Larcoche J. Claverol S. Carde J. P. Hartmann M. A.. (2004). Lipid rats in higher plant cells: Puriication and characterization of Triton X-100-insoluble microdomains from tobacco plasma membrane. J Biol Chem 279, 36277–36286. doi: 10.1074/jbc.M403440200 15190066

[B40] Nohzadeh M. S. Habibi R. M. Heidari M. Salekdeh G. H. (2007). Proteomics reveals new salt responsive proteins associated with rice plasma membrane. Biosci. Biotech. Bioch. 71 (9), 2144–2154. doi: 10.1271/bbb.70027 17827676

[B41] Perraki A. Cacas J. L. Crowet J. M. Lins L. Castroviejo M. German-Retana S.. (2012). Plasma membrane localization of solanum tuberosum remorin from group 1, homolog 3 is mediated by conformational changes in a novel c-terminal anchor and required for the restriction of potato virus X movement. Plant Physiol. 160 (2), 624–637. doi: 10.1104/pp.112.200519 22855937 PMC3461544

[B42] Raffaele S. Mongrand S. Gamas P. Niebel A. Ott T. (2007). Genome-wide annotation of remorins, a plant-specific protein family: evolutionary and functional perspectives. Plant Physiol. 145 (3), 593–600. doi: 10.1104/pp.107.108639 17984200 PMC2048807

[B43] Raffaele S. Perraki A. Mongrand S. (2013). The remorin c-terminal anchor was shaped by convergent evolution among membrane binding domains. Plant Signal Behav. 8 (3), e23207. doi: 10.4161/psb.23207 23299327 PMC3676492

[B44] Reddy A. R. Ramakrishna W. Sekhar A. C. Ithal N. Babu P. R. Bonaldo M. F.. (2002). Novel genes are enriched in normalized cDNA libraries from drought-stressed seedlings of rice (*Oryza sativa* l. subsp. *indica* cv. nagina 22). Genome. 45 (1), 204–211. doi: 10.1139/g01-114 11908663

[B45] Reymond P. Kunz B. Paul-Pletzer K. Grimm R. Eckerskorn C. Farmer E. E. (1996). Cloning of a cDNA encoding a plasma membrane-associated, uronide binding phosphoprotein with physical properties similar to viral movement proteins. Plant Cell. 8 (12), 2265–2276. doi: 10.1105/tpc.8.12.2265 8989883 PMC161351

[B46] Rogozin I. B. Sverdlov A. V. Babenko V. N. Koonin E. V. (2005). Analysis of evolution of exon-intron structure of eukaryotic genes. Brief Bioinform. 6 (2), 118–134. doi: 10.1093/bib/6.2.118 15975222

[B47] Tóth K. Stratil T. F. Madsen E. B. Ye J. Popp C. Antolín-Llovera M.. (2012). Functional domain analysis of the remorin protein LjSYMREM1 in *Lotus japonica* s. PloS One 7 (1), e30817. doi: 10.1371/journal.pone.0030817 22292047 PMC3264624

[B48] Voorrips R. E. (2002). MapChart: software for the graphical presentation of linkage maps and QTLs. J. Heredity. 93 (1), 77–78. doi: 10.1093/jhered/93.1.77 12011185

[B49] Wai A. H. Naing A. H. Lee D. J. Kim C. K. Chung M. Y. (2020). Molecular genetic approaches for enhancing stress tolerance and fruit quality of tomato. Plant Biotechnol. Rep. 14, 515–537. doi: 10.1007/s11816-020-00638-1

[B50] Wang Y. Li J. Li M. Li Y. Zhao Z. Li C.. (2022). Genome-wide characterization of remorin genes in terms of their evolution and expression in response to hormone signals and abiotic stresses in foxtail millet *(Setaria italica*). Diversity. 14, 711. doi: 10.3390/d14090711

[B51] Wang Y. Tang H. Debarry J. D. Tan X. Li J. Wang X.. (2012). MCScanX: a toolkit for detection and evolutionary analysis of gene synteny and collinearity. Nucleic Acids Res. 40 (7), e49. doi: 10.1093/nar/gkr1293 22217600 PMC3326336

[B52] Xiong E. Zheng C. Wu X. Wang W. (2015). Protein subcellular location: the gap between prediction and experimentation. Plant Mol. Biol. Rep. 34 (1), 1–10. doi: 10.1007/s11105-015-0898-2

[B53] Yadav C. B. Bonthala V. S. Muthamilarasan M. Pandey G. Khan Y. Prasad M. (2015). Genome-wide development of transposable elements-based markers in foxtail millet and construction of an integrated database. DNA Res. 22 (1), 79–90. doi: 10.1093/dnares/dsu039 25428892 PMC4379977

[B54] Yue J. Li C. Liu Y. Yu J. (2014). A remorin gene SiREM6, the target gene of SiARDP, from foxtail millet *(Setaria italica*) promotes high salt tolerance in transgenic *Arabidopsis* . PloS One 9 (6), e100772. doi: 10.1371/journal.pone.0100772 24967625 PMC4072699

