# Supplementary material for: Molecular characterization and expression analysis of the remorin genes in tomato (Solanum lycopersicum L.)
Source: Front Plant Sci. 2023 May 9;14:1175153. doi: 10.3389/fpls.2023.1175153 (PMC10203495; doi:10.3389/fpls.2023.1175153)
Supplement: Supplementary file 2 [file Table_2.docx]

**Supplementary Table 2 Homologous *REM* gene pairs between tomato and *Arabidopsis.***

| Homologous *SlREM* gene | Homologous *AtREM* gene |
| --- | --- |
| *SlREM2* | *AtREM12* |
| *SlREM3* | *AtREM14* |
| *SlREM4* | *AtREM14* |
| *SlREM5* | *AtREM10* |
| *SlREM5* | *AtREM15* |
| *SlREM6* | *AtREM4* |
| *SlREM7* | *AtREM6* |
| *SlREM7* | *AtREM1* |
| *SlREM8* | *AtREM11* |
| *SlREM8* | *AtREM8* |
| *SlREM10* | *AtREM6* |
| *SlREM10* | *AtREM1* |
| *SlREM11* | *AtREM5* |
| *SlREM17* | *AtREM11* |
| *SlREM17* | *AtREM8* |
