# Supplementary material for: Molecular characterization and expression analysis of the remorin genes in tomato (Solanum lycopersicum L.)
Source: Front Plant Sci. 2023 May 9;14:1175153. doi: 10.3389/fpls.2023.1175153 (PMC10203495; doi:10.3389/fpls.2023.1175153)
Supplement: Supplementary file 3 [file Table_3.docx]

**Supplementary Table 3 Selective pressure analysis of *REM* genes family.**

| **species** | **A pair of genes** | | **S** | **N** | **ds** | **d_N_** | **d_N_/d_s_** |
| --- | --- | --- | --- | --- | --- | --- | --- |
| **tomato** | | *SlREM5*-*SlREM13* | 127.97 | 418.04 | 0.741237 | 0.23297 | 0.3143 |
|  |  | *SlREM8*-*SlREM17* | 149.91 | 576.10 | 0.995835 | 0.92205 | 0.9259 |
|  |  | *SlREM3*-*SlREM4* | 257.72 | 861.29 | 0.735793 | 0.22188 | 0.30156 |
|  |  | *SlREM7*-*SlREM10* | 217.76 | 667.24 | 0.877689 | 0.38895 | 0.44315 |
| **tomato and *Arabidopsis*** | | *AtREM1*-*SlREM10* | 261.26 | 696.75 | 1.61943 | 0.643729 | 0.397505 |
|  |  | *AtREM1*-*SlREM7* | 181.77 | 583.23 | 0.618794 | 1.11871 | 1.80805 |
|  |  | *AtREM4*-*SlREM6* | 220.02 | 736.98 | 2.434 | 0.571869 | 0.234951 |
|  |  | *AtREM5*-*SlREM11* | 168.56 | 635.44 | 1.18623 | 0.9506 | 0.801364 |
|  |  | *AtREM5*-*SlREM10* | 63.66 | 236.34 | 2.96449 | 0.470811 | 0.158817 |
|  |  | *AtREM6*-*SlREM7* | 60.39 | 266.61 | 1.06778 | 0.984648 | 0.922143 |
|  |  | *AtREM8*-*SlREM8* | 219.61 | 437.40 | 0.328899 | 0.825008 | 2.5084 |
|  |  | *AtREM8*-*SlREM17* | 283.84 | 1045.16 | 1.00785 | 0.971078 | 1.03787 |
|  |  | *AtREM10*-*SlREM5* | 119.6 | 336.4 | 0.843907 | 1.0555 | 1.25073 |
|  |  | *AtREM11*-*SlREM8* | 156.24 | 554.76 | 0.81995 | 1.05071 | 1.28143 |
|  |  | *AtREM11*-*SlREM17* | 266.14 | 1026.86 | 0.863264 | 1.03544 | 1.19945 |
|  |  | *AtREM12*-*SlREM2* | 134.31 | 378.69 | 2.80869 | 0.385499 | 0.127639 |
|  |  | *AtREM14*-*SlREM4* | 215.89 | 789.16 | 1.06515 | 0.982176 | 0.922099 |
|  |  | *AtREM14*-*SlREM3* | 254.10 | 786.90 | 1.47535 | 0.647482 | 0.438866 |
|  |  | *AtREM15*-*SlREM5* | 131.04 | 447.96 | 1.84345 | 0.196018 | 0.106332 |
