# Supplementary material for: Molecular characterization and expression analysis of the remorin genes in tomato (Solanum lycopersicum L.)
Source: Front Plant Sci. 2023 May 9;14:1175153. doi: 10.3389/fpls.2023.1175153 (PMC10203495; doi:10.3389/fpls.2023.1175153)
Supplement: Supplementary file 4 [file Table_4.docx]

**Supplementary Table 4 *SlREM* genes used to construct the phylogenetic tree.**

| Species | Gene names | Gene ID | Database |
| --- | --- | --- | --- |
| *Arabidopsis thaliana* | *AtREM1.1* | *AT3G48940* | (*Raffaele S, et al., 2007*) |
|  | *AtREM1.2* | *AT3G61260* |  |
|  | *AtREM1.3* | *AT2G45820* |  |
|  | *AtREM1.4* | *AT5G23750* |  |
|  | *AtREM3.1* | *AT1G69325* |  |
|  | *AtREM3.2* | *AT4G00670* |  |
|  | *AtREM4.1* | *AT3G57540* |  |
|  | *AtREM4.2* | *AT2G41870* |  |
|  | *AtREM5.1* | *AT1G45207* |  |
|  | *AtREM6.1* | *AT2G02170* |  |
|  | *AtREM6.2* | *AT1G30320* |  |
|  | *AtREM6.3* | *AT1G53860* |  |
|  | *AtREM6.4* | *AT4G36970* |  |
|  | *AtREM6.5* | *AT1G67590* |  |
|  | *AtREM6.6* | *AT1G13920* |  |
|  | *AtREM6.7* | *AT5G61280* |  |
|  |  |  |  |
| *Medicago truncatula* | *MtREM1.1* | *MtC60319* | (*Raffaele S, et al., 2007*) |
|  | *MtREM1.2* | *MtC00278* |  |
|  | *MtREM1.3* | *MtD17655* |  |
|  | *MtREM2.1* | *MtD05125* |  |
|  | *MtREM2.2* | *MtC10811* |  |
|  | *MtREM3.1* | *ABE84731* |  |
|  | *MtREM3.2* | *MtD26156* |  |
|  | *MtREM4.1* | *MtC93157* |  |
|  | *MtREM5.1* | *MtD03479* |  |
|  | *MtREM5.2* | *MtD17032* |  |
|  |  |  |  |
| *Oryza sativa* | *OsREM0.2* | *Os10g17790* | (*Raffaele S, et al., 2007*) |
|  | *OsREM1.1* | *Os02g57840* |  |
|  | *OsREM1.2* | *Os10g36000* |  |
|  | *OsREM1.3* | *Os03g02040* |  |
|  | *OsREM1.4* | *Os02g42880* |  |
|  | *OsREM1.5* | *Os04g45070* |  |
|  | *OsREM4.1* | *Os07g38170* |  |
|  | *OsREM4.2* | *Os03g59360* |  |
|  | *OsREM4.3* | *Os07g10780* |  |
|  | *OsREM5.1* | *Os02g52810* |  |
|  | *OsREM5.2* | *Os08g36760* |  |
|  | *OsREM5.3* | *Os09g28300* |  |
|  | *OsREM6.1* | *Os02g02500* |  |
|  | *OsREM6.2* | *Os03g02840* |  |
|  | *OsREM6.3* | *Os12g41940* |  |
|  | *OsREM6.4* | *Os11g40210* |  |
|  | *OsREM6.5*  *OsREM6.6* | *Os02g39000*  *Os04g52920* |  |
|  |  |  |  |
| *Zea mays* | *ZmREM1.1* | *TA104935_4577* | (*Raffaele S, et al., 2007*) |
|  | *ZmREM1.2* | *CF004505* |  |
|  | *ZmREM4.1* | *TA114130_4577* |  |
|  | *ZmREM4.2* | *TA119747_4577* |  |
|  | *ZmREM6.1* | *TA117003_4577* |  |
|  | *ZmREM6.2* | *TA135644_4577* |  |
|  |  |  |  |
| *Solanum lycopersicum* | *SlREM1* | *Solyc01g008180.3* | Ensembl |
|  | *SlREM2* | *Solyc01g094370.3* |  |
|  | *SlREM3* | *Solyc02g064990.3* |  |
|  | *SlREM4* | *Solyc02g090900.3* |  |
|  | *SlREM5* | *Solyc03g025850.3* |  |
|  | *SlREM6* | *Solyc03g123590.3* |  |
|  | *SlREM7* | *Solyc04g005470.3* |  |
|  | *SlREM8* | *Solyc04g015520.3* |  |
|  | *SlREM9* | *Solyc04g078810.3* |  |
|  | *SlREM10* | *Solyc05g012550.3* |  |
|  | *SlREM11* | *Solyc05g014710.3* |  |
|  | *SlREM12* | *Solyc05g048820.3* |  |
|  | *SlREM13* | *Solyc06g035920.3* |  |
|  | *SlREM14* | *Solyc06g069590.3* |  |
|  | *SlREM15* | *Solyc08g045640.3* |  |
|  | *SlREM16* | *Solyc10g017540.3* |  |
|  | *SlREM17* | *Solyc10g080220.2* |  |
