# Supplementary material for: Molecular characterization and expression analysis of the remorin genes in tomato (Solanum lycopersicum L.)
Source: Front Plant Sci. 2023 May 9;14:1175153. doi: 10.3389/fpls.2023.1175153 (PMC10203495; doi:10.3389/fpls.2023.1175153)
Supplement: Supplementary file 5 [file Table_5.docx]

**Supplementary Table 5 Conserved motifs in the amino acid sequence of SlREM proteins**

| **Motif** | **Width Multilevel** | **Consensus sequence** |
| --- | --- | --- |
| 1 | 41 | WEESEKSKVNNKYKREEAKIQAWENLKKAKAEAELKKLEEK |
| 2 | 41 | MKNKIALIRKKAEEKRASAEAKRGEELLKVEEKAAKIRATG |
| 3 | 8 | SFSGCFWC |
| 4 | 21 | FDRDAALAQLETEKRSSFIKA |
| 5 | 14 | GFQKGWSSERVPLP |
| 6 | 29 | ALMPYNSGRPLPSKWDDAEKWIVSPVSGY |
| 7 | 66 | NVQPQRRPKSKSGPLGPPGLMYLPNYSPSVPVFEGGNGSNFIANSPFTTGVLVPDGLSIHYGAGEG |
| 8 | 26 | RSVSRRDMGTZMTPIASQEPSPTATP |
| 9 | 72 | PNKVTLVGVLKACAISGALDEGRRVHAHIIQSGIENSLELQTALVNLYAKCGYIDKAYEVFEAMPFKBAPAW |
| 10 | 86 | RDRGIPPQKTQLFKETKRIPSWLERQFPRKASRDYDSSDSIDYPAAVAVAAFVIKSIEEKSEKDQRKTNIGGDKPLSKIKSKGEDI |
