# Supplementary material for: Molecular characterization and expression analysis of the remorin genes in tomato (Solanum lycopersicum L.)
Source: Front Plant Sci. 2023 May 9;14:1175153. doi: 10.3389/fpls.2023.1175153 (PMC10203495; doi:10.3389/fpls.2023.1175153)
Supplement: Supplementary file 6 [file DataSheet_1.docx]

Supplementary**
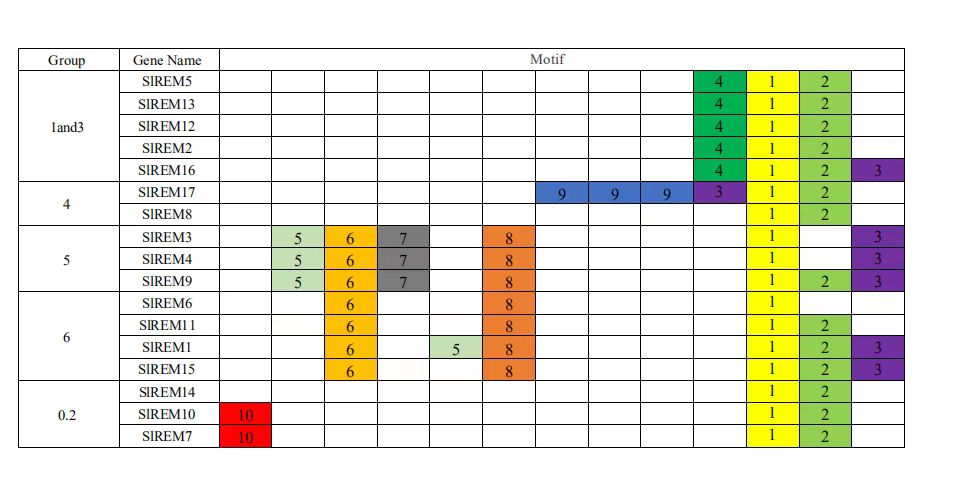
** Figure 1 Schematic diagram of the conserved motifs in 17 tomato remorin (REM) genes. The motifs were identified by MEME software. Each colored number represents a motif. The remorin C-domain represents Motif 1, Motif 2 and Motif 3, which are shown in yellow, light green and purple box, respectively. The remorin N-domain represents Motif 4. The other motifs are shown in different colors.


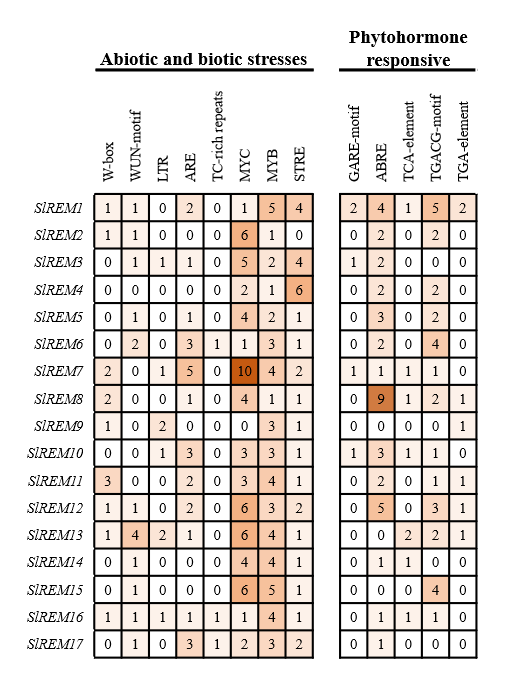


Supplementary Figure 2 Cis-acting element analysis of the *SlREM* gene family in tomato. Numbers in the box are the number of cis-elements indicated by different intensity colors and numbers.
